# CHD4 as an important mediator in regulating the malignant behaviors of colorectal cancer

**DOI:** 10.7150/ijbs.56976

**Published:** 2021-04-12

**Authors:** Chia-Lo Chang, Chi-Ruei Huang, Shu-Jyuan Chang, Chun-Chieh Wu, Hong-Hwa Chen, Chi-Wen Luo, Hon-Kan Yip

**Affiliations:** 1 Division of Colorectal Surgery, Department of Surgery, Kaohsiung Chang Gung Memorial Hospital and Chang Gung University College of Medicine, Kaohsiung, Taiwan.; 2 Center for Shockwave Medicine and Tissue Engineering, Kaohsiung Chang Gung Memorial Hospital, Kaohsiung, Taiwan.; 3 Division of Cardiology, Department of Internal Medicine, Kaohsiung Chang Gung Memorial Hospital and Chang Gung University College of Medicine, Kaohsiung, Taiwan.; 4 Department of Pathology, Kaohsiung Medical University Hospital, Kaohsiung, Taiwan.; 5 Department of Pathology, College of Medicine, Kaohsiung Medical University, Kaohsiung, Taiwan.; 6 Department of Surgery, Kaohsiung Medical University Hospital, Kaohsiung, Taiwan.; 7 Division of Breast Surgery, Department of Surgery, Kaohsiung Medical University Hospital, Kaohsiung, Taiwan.; 8 Institute for Translational Research in Biomedicine, Kaohsiung Chang Gung Memorial Hospital Kaohsiung, Taiwan.; 9 Department of Medical Research, China Medical University Hospital, China Medical University, Taichung, Taiwan.; 10 Department of Nursing, Asia University Taichung, Taiwan.; 11 Division of Cardiology, Department of Internal Medicine, Xiamen Chang Gung Hospital, Xiamen, Fujian, China.

**Keywords:** colorectal cancer, CHD4, metastasis, drug resistance

## Abstract

Colorectal cancer (CRC) has ranked first in terms of incidence in Taiwan. Surgical resection combined with chemo-, radio-, or targeted-therapies are the main treatments for CRC patients in current clinical practice. However, many CRC patients still respond poorly to these treatments, leading to tumor recurrence and an unacceptably high incidence of metastasis and death. Therefore, appropriate diagnosis, treatment, and drug selection are pressing issues in clinical practice.

The Mi-2/nucleosome remodeling and deacetylase complex is an important epigenetic regulator of chromatin structure and gene expression. An important component of this complex is chromodomain-helicase-DNA-binding protein 4 (CHD4), which is involved in DNA repair after injury. Recent studies have indicated that CHD4 has oncogenic functions that inhibit multiple tumor suppressor genes through epigenetic regulation. However, the role of CHD4 in CRC has not yet been well investigated.

In this study, we compared CHD4 expression in CRC patients from The Cancer Genome Atlas database. We found higher levels of CHD4 expression in CRC patients. In a series of *in vitro* experiments, we found that CHD4 affected cell motility and drug sensitivity in CRC cells. In animal models, the depletion of CHD4 affected CRC tumor growth, and the combination of a histone deacetylase 1 (HDAC1) inhibitor and platinum drugs inhibited CHD4 expression and increased the cytotoxicity of platinum drugs. Moreover, CHD4 expression was also a prognostic biomarker in CRC patients.

Based on the above results, we believe that CHD4 expression is a viable biomarker for predicting metastasis CRC patients, and it has the potential to become a target for drug development.

## Introduction

Colorectal cancer (CRC) represents 15% of all cancers worldwide 1. CRC is a multistep disorder caused by the accumulation of genetic and epigenetic aberrations under microenvironmental influence. Gene abnormalities, such as those in *p53*, *WNT*, DNA mismatch repair genes, and *RAS*, have been considered to drive the development of a benign adenoma to a carcinoma or metastatic disease 2, 3. Previous studies have suggested that heritable genetic changes and genomic instability also played important roles in CRC development. In addition, chronic inflammation was considered a risk factor for the CRC development and metastasis 4. CRC occurrence have also been linked to several environmental factors 5. In recent years, epigenetic mechanisms were found to record the effects of environmental challenges on the genome level. Therefore, they play important roles in the pathogenesis of inflammation-associated CRC 6. Despite advancements in drug development and diagnostic methods, CRC is still the third leading cause of cancer-related deaths worldwide 7. Thus, increasing the survival rate of CRC remains a major challenge for medical institutions worldwide.

Many investigators have studied epigenetic alterations in the CRC cell genome and cancer development over the last two decades 8-11. Epigenetic aberrations play important roles in affecting every aspect of tumor development process from tumor initiation to metastasis 3. Although multiple epigenetic mechanisms are involved in the pathogenesis of different cancer types and cross-interact with one other, DNA methylation and histone modifications in the gene promoter regions are the most extensively studied mechanisms. These were also suggested to be the main mediators of CRC epigenetic inheritance in cancer cells 12, 13. Ongoing studies have focused on the potential role of recently discovered epigenetic regulators in the treatment of cancer patients and their potential prognostic and predictive values 14.

The nucleosome remodeling and deacetylase (NuRD) complex has been suggested to play important roles in regulating chromatin remodeling, gene expression, and cell cycle progression in normal development and tumorigenesis 15-17. Among the components of the NuRD complex, chromodomain helicase DNA binding protein 4 (CHD4) is the largest subunit. It is involved in the regulation of cell cycle and DNA repair 18-20. A prior study also suggested that CHD4 controlled homologous recombination repair to maintain genome stability. Another study indicated that CHD4 could initiate and maintain the epigenetic suppression of multiple tumor suppressor genes, implying that CHD4 has oncogenic functions 21. CHD4 deficiency has potential therapeutic implications in tumors 22. Knockdown of CHD4 could overcome the resistance of approved drugs in acute myeloid leukemia cells or in human epidermal growth factor receptor 2-positive patients 23, 24 and also inhibit tumor growth and increase cytotoxicity in breast cancer 24-27. Together with CHD4, histone deacetylase 1 (HDAC1) and HDAC2 are the core subunits of the NuRD complex. These interact to maintain the silencing of tumor suppressor genes (TSGs). Depletion of CHD4 is synergistic with DNA methyltransferase inhibition in reducing cancer cell viability. In relation to the reactivation of tumor suppressor genes, the combined inhibition of these proteins may be beneficial for cancer treatment 28. However, another study indicated that CHD4 was a pan-cancer biomarker to another HDAC inhibitor sensitivity in colon and breast cancer cells 29. Therefore, it is necessary to understand the role of CHD4 in regulating the malignant characteristics of CRC.

In this study, we aimed to clarify the role of CHD4 in CRC, to explore the feasibility of using CHD4 as a prognostic biomarker in CRC patients, and to elucidate whether CHD4 could be a target for future anticancer drug development for CRC patients.

## Materials and Methods

### Cell lines

Colorectal cancer cell lines DLD-1, HCT-116, and HT-29 (ATCC, Manassas, VA, USA) were used in this study. Cells were maintained in Dulbecco's modified Eagle's medium (DMEM) supplemented with 10% fetal bovine serum (FBS) (Hyclone Laboratories Inc., South Logan, UT, USA) and antibiotics at 37 °C in a humidified atmosphere with 5% CO_2_.

### Antibodies

Antibodies against CHD4 (GTX124186) were purchased from GeneTex Inc. (Hsinchu City, Taiwan, R.O.C). Antibodies against Caspase 3 (#9662S), cleaved poly (ADP-ribose) polymerase (PARP) (#9541), Vimentin (#5741), N-cadherin (#5296), E-cadherin (#13116), matrix metalloproteinase (MMP)-2 (#4022), p-γH2A.X (#9718), GAPDH (#2118) and Actin (#8480) were purchased from Cell Signaling Technology (Beverly, MA, USA). Mouse (GTX213111) and rabbit (GTX213110) IgG (HRP) antibodies from GeneTex Inc. were used as the secondary antibodies.

### Immunoblotting

Protein extraction and immunoblotting were performed as previously described 30, 31. In brief, cells were lysed in the protein extraction buffer (Thermo Scientific, Rockford, IL, USA). Then, the proteins were collected, loaded on a sodium dodecyl sulfate-polyacrylamide gel, and transferred to nitrocellulose. Immunoblotting was performed using primary and secondary antibodies.

### *CHD4* short interfering RNA transfection

Short interfering RNA (siRNA) for human *CHD4* (L-009774-00-0005) and negative control (CN-001000-01-05) were purchased from Dharmacon (Dharmacon Life Technologies, Cologne, Germany). Appropriate non-targeting and specific siRNA were transfected to cells following to the manufacturer's protocol (Thermo, Carlsbad, CA, USA).

### *CHD4* shRNA clone transfection

shRNA clones were obtained from the National RNAi Core Facility Platform from Academia Sinica, Taiwan. Individual clones were identified by their unique TRC number (e.g., shCHD4: TRCN0000021363, shLacZ: TRCN0000072233). Transient transfection of cells with plasmids was performed following the manufacture's protocol. Transfected cells were selected using puromycin, and the efficiency of *CHD4* silencing was evaluated using real-time reverse transcription-polymerase chain reaction.

### Cell proliferation assay

Cells (1 × 10^4^) were seeded in each well of a 24-well plate. After treatment with different doses of platinum drugs for at least 72 h, the cells were stained with 3-(4,5-dimethylthiazol-2-yl)-2,5-diphenyltetrazolium bromide (MTT) solution (Sigma, St. Louis, MO, USA) and incubated for 1.5 h. Formazan crystals were solubilized in dimethyl sulfoxide (Sigma, St. Louis, MO, USA) and measured at 590 nm. In addition, the apoptotic cell death was evaluated using staining with FITC Annexin V apoptosis detection kit (BD Biosciences, San Jose, CA, USA) and detected using flow cytometry (LSR II Flow Cytometer, BD Biosciences). Each experiment was repeated at least three times.

### Wound healing assay

Cells were seeded in two-well silicone inserts (ibidi GmBH, Planegg, Germany) and cultured in the culture medium at 37 °C in a 5% CO_2_ incubator for 24 h. Then, the inserts were removed. Images were then captured at 0 to 48 h. Each experiment was repeated at least three times.

### Transwell cell invasion assay

Cell invasion assays were performed as previously described 32. Cells were seeded in the upper insert chamber which pre-coated with Matrigel and containing serum-free medium. A culture medium was added to the bottom chamber. After 24 h of incubation period, cells were rinsed and stained with Giemsa solution (Sigma, St. Louis, MO, USA), and the number of invading cells was counted. Each experiment was repeated at least three times.

### Ethics statement

Animal experimental procedures were approved by the Institute of Animal Care and Use Committee at Kaohsiung Chang Gung Memorial Hospital (Affidavit of Approval of Animal Use Protocol No. 2017110301) and performed in accordance with the Guide for the Care and Use of Laboratory Animals. Animals were housed in an Association for Assessment and Accreditation of Laboratory Animal Care International (Frederick, MD, USA)-approved animal facility in Kaohsiung Chang Gung Memorial Hospital.

### Animal grouping, time courses of Oxaliplatin and SAHA treatment, and tumor size measurement

The pathogen-free, 12-week-old male nude mice (Charles River Technology, BioLASCO, Taiwan) were used in this study. We equally separated these mice into four groups. Group 1: DLD-1 cells (2.0 × 10^6^ cells) were subcutaneously injected to the back of nude mice without treating any drugs; Group 2: shCHD4 DLD-1 cells (2.0 × 10^6^ cells) were subcutaneously injected to the back of nude mouse without treating with any drugs; Group 3: DLD-1 cells (2.0 × 10^6^ cells) were subcutaneously injected to the back of nude mice and intra-peritoneal injected with oxaliplatin (12 mg/kg/week) from day 14 to 32; Group 4: DLD-1 cells (2.0 × 10^6^ cells) were subcutaneously injected to the back of nude mice and intra-peritoneally injected with oxaliplatin (12 mg/kg/week) and suberoylanilide hydroxamic acid** (**SAHA) (50 mg/kg/day) from day 14 to day 32. Tumor growth was measured using the following formula: tumor volume = (width^2^× length)/2 after the implantation of the tumor cells. Tumors were removed and measured after euthanizing the animals at day 32 post-implantation. Tumor specimens were used for immunohistochemical (IHC) staining and protein extraction. The dosage of oxaliplatin and SAHA was based on previous studies 33, 34 with some modifications.

### Human samples

A total of 40 paired CRC tissue specimens were collected in the Tissue Bank, Kaohsiung Chang Gung Memorial Hospital. All participants provided written informed consent at the time of recruitment. All cases were confirmed with diagnoses of colorectal cancer clinically and pathologically based on the revised international system for staging of colorectal cancer and tissues were kept at -80°C for storage. The Institutional Review Board approval for using these human tissues in this study was given by the Research Ethics Committee of the Kaohsiung Chang Gung Memorial Hospital (IRB-201701900B0) on 25 Nov 2016.

### Immunohistochemistry staining and scoring

Colorectal cancer tissue microarrays (SuperBioChips Laboratories, Gangnam-gu, KR) were used in this study. The details on clinicopathological and survival information of patients were provided on the manufacturer's websites. To perform the immunohistochemistry (IHC) staining, paraffin embedded tissue sections were de-paraffinized, rehydrated, retrieval, treated with H_2_O_2_ and then incubated with primary antibodies specifically against CHD4, appropriate secondary antibody and the Envision system (Dako, Denmark) 26. Finally, sections were counterstained with hematoxylin and then analyzed by microscope. CHD4 expression has been defined as the nucleus staining in tumor cells and CHD4 expression in different patients was scored based on the product of signal intensity and the proportion of positive cells 25, 26, 35.

### Statistical analysis

CHD4 expression determined using IHC staining in CRC tissues was compared with patient survival and assessed using the Chi-square test. To evaluate the significance of CHD4 in CRC prognosis, survival curves were obtained using the Kaplan-Meier method. In addition, the difference between each group was compared and calculated by two-tailed Student's *t*-test. *P* < 0.05 was considered statistically significant. All statistical analyses were performed using SPSS 19.0 software (IBM Corp., Armonk, NY, USA).

## Results

### Associations between CHD4 and clinicopathological parameters in CRC patients

**Figure 1:**
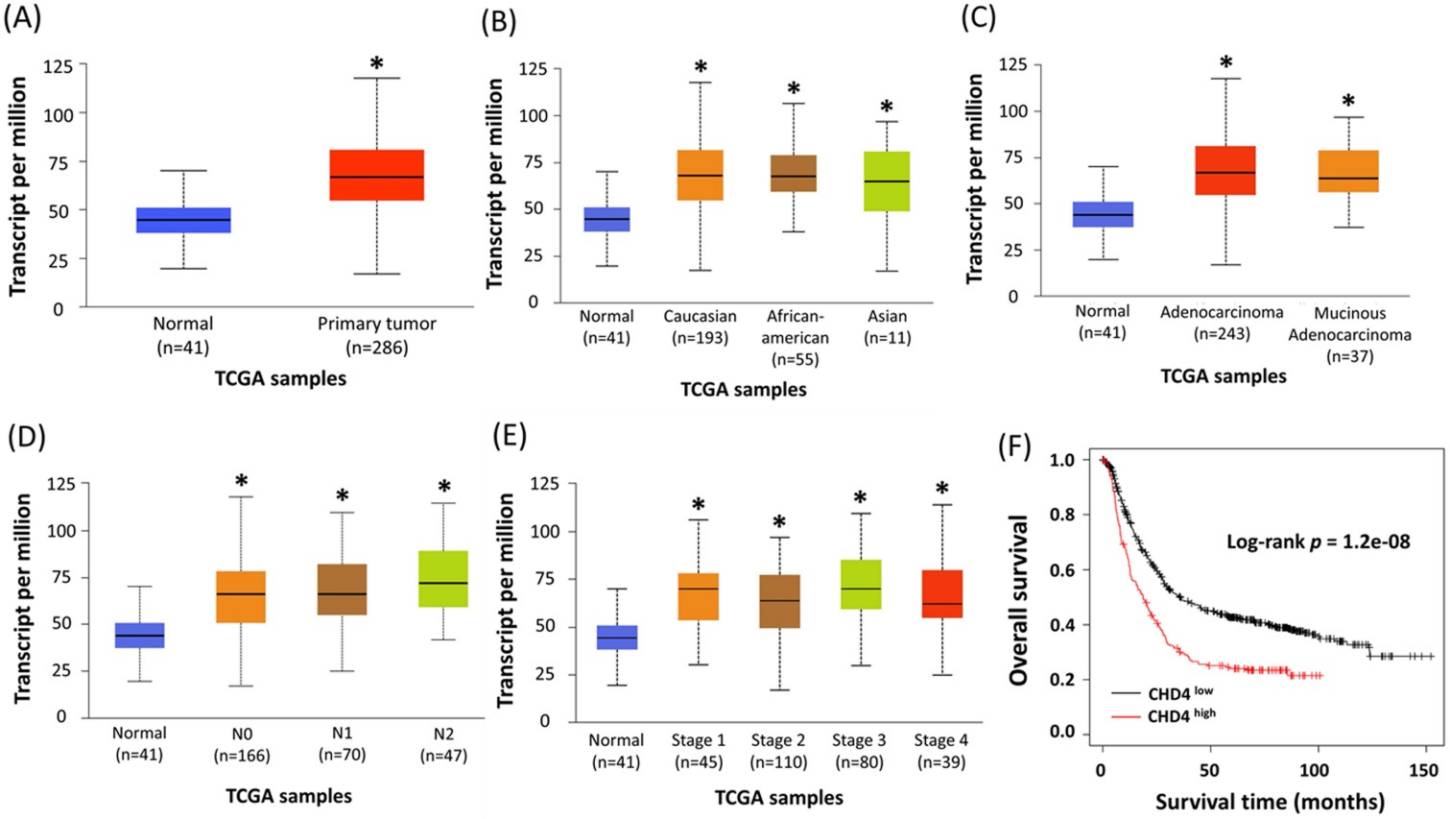
** Higher expression of *CHD4* mRNA is associated with poor prognosis in CRC patients.** (A-E) Patients with CRC express significantly higher *CHD4* mRNA than healthy individuals (A). This tendency is observed regardless of patient ethnicity (Caucasian, African-American, and Asian) (B), histological subtypes (such as adenocarcinoma and mucinous adenocarcinoma) (C), nodal metastasis (N0 to N2) (D), and individual cancer stages (E). All patients with CRC express significantly higher level of *CHD4* mRNA than healthy individuals. These data are obtained from UALCAN database in TCGA CRC cancer samples. (F) Kaplan-Meier analyses of overall survival in CRC patients from online KM plotter. Patients are stratified into 'low' and 'high' *CHD4* mRNA expression based on auto select best cutoff (The highest HR value (or 1/HR in case of HR<1) in case of *P* = 0 in the Cox regression). *P < 0.05. HR: Hazard ratio.

CHD4 has been reported as an important regulator in several cancer types. However, its role in CRC is still unclear. To assess the role of CHD4 in CRC patients, we used colon cancer samples from the Ualcan database by The Cancer Genome Atlas to analyze the association between *CHD4* mRNA expression and several clinical parameters. Our results showed that regardless of patient ethnicity (Caucasian, African-American, and Asian), histological subtypes (such as adenocarcinoma and mucinous adenocarcinoma), nodal metastasis (N0 to N2), TP53 mutation status, and individual cancer stages, CRC patients expressed significantly higher *CHD4* mRNA expression than healthy individuals (Fig. 1A-E). The Kaplan-Meier plotter database showed that high *CHD4* mRNA expression was also correlated with short overall survival (Fig. 1F). Thus, CHD4 plays an important role in regulating the malignant characteristics of CRC.

### CHD4 protein expression is usually overexpressed in most CRC patients and correlates with patient survival

**Figure 2:**
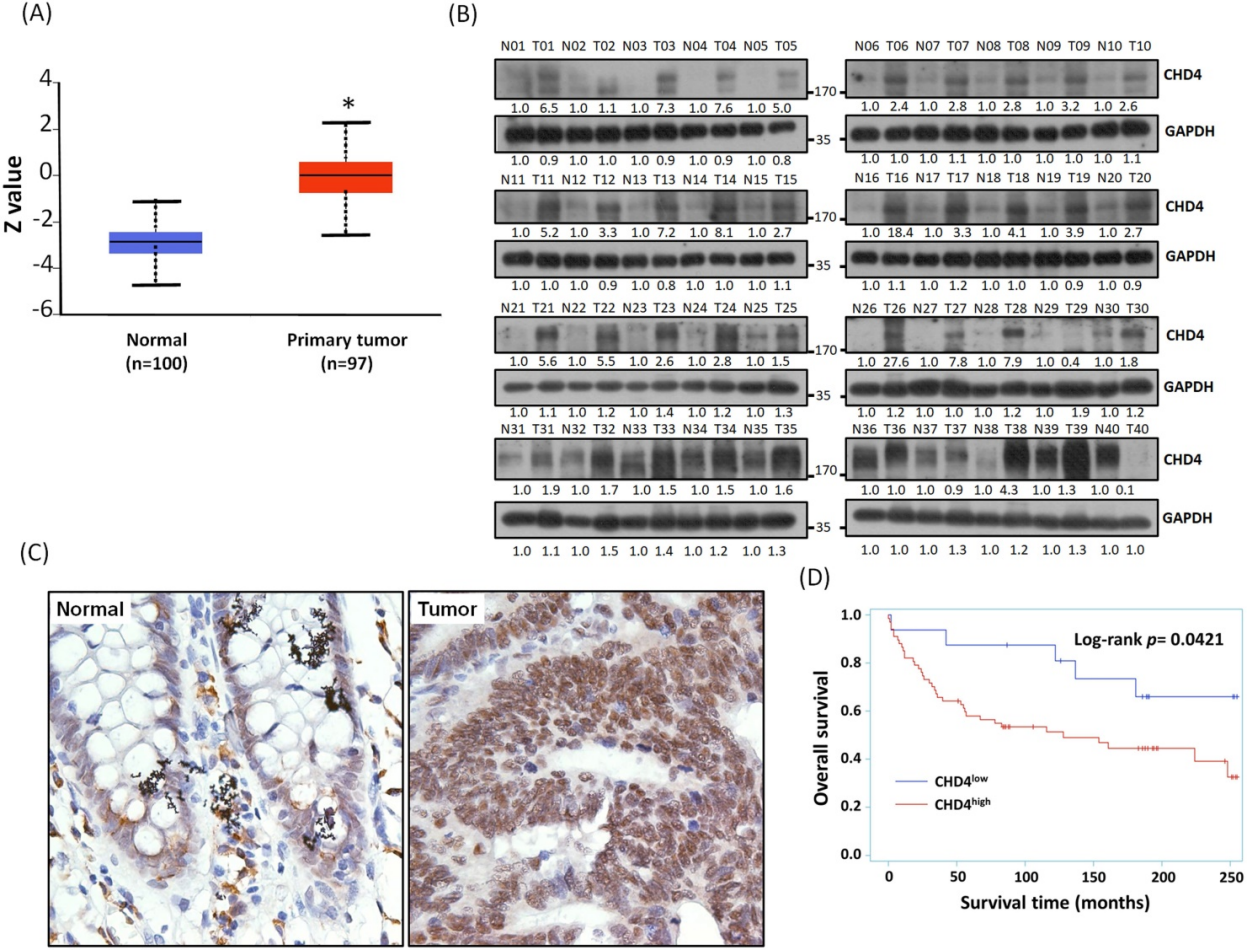
** Representative immunohistochemistry staining results and protein expression of CHD4 in tumor cells of CRC patients.** (A) Protein expression of CHD4 in normal and tumor tissues analyzed by CPTAC in CRC patients. (B) CHD4 protein expression in 40 CRC patients. (C) CHD4 expression in normal and tumor tissue in CRC patients (original 400x magnification). The classification of CHD4 expression was according to the staining observed in the cell nucleus. (D) Kaplan-Meier survival curves in CRC patients.

We have found that the mRNA expression of *CHD4* was highly expressed in CRC patients from the Ualcan database. In addition, the result of Clinical Proteomic Tumor Analysis Consortium (CPTAC) analysis also showed that CHD4 protein expression in CRC tissues was also higher than it in normal tissues (Fig. 2A). In this study, we collected 40 CRC patient samples in our hospital and analyzed CHD4 protein expression. As shown in Fig. 2B, most paired samples from the same CRC patients showed that CHD4 protein expression was higher in tumor tissues than in adjacent non-tumor colon epithelial tissues (About 85% of CHD4 protein expression in tumor tissues were at least 1.5 times higher than adjacent non-tumor colon epithelial tissues), suggested that CHD4 could be a diagnostic marker in CRC cancer. Next, we used the tissue microarrays (TMA) to investigate the relationships between CHD4 and several clinical parameters in CRC patients. Fig. 2C shows the representative IHC staining results of the nuclear levels of CHD4 in adjacent non-tumor colon epithelial tissue and tumor tissue from CRC patients. CHD4 expression was higher in the nuclei of tumor cells than in normal colon epithelial cells. Table 1 indicates that CHD4 expression was significantly associated with the metastatic stage and survival. Kaplan-Meier analysis also showed that the survival duration in patients with high CHD4 levels was significantly lower than that in patients with low CHD4 expression (Fig. 2D). Thus, CHD4 has the potential to be an important diagnostic and prognostic biomarker in CRC patients.

### CHD4 affects CRC cell malignant characteristics such as cell proliferation and motility

**Figure 3:**
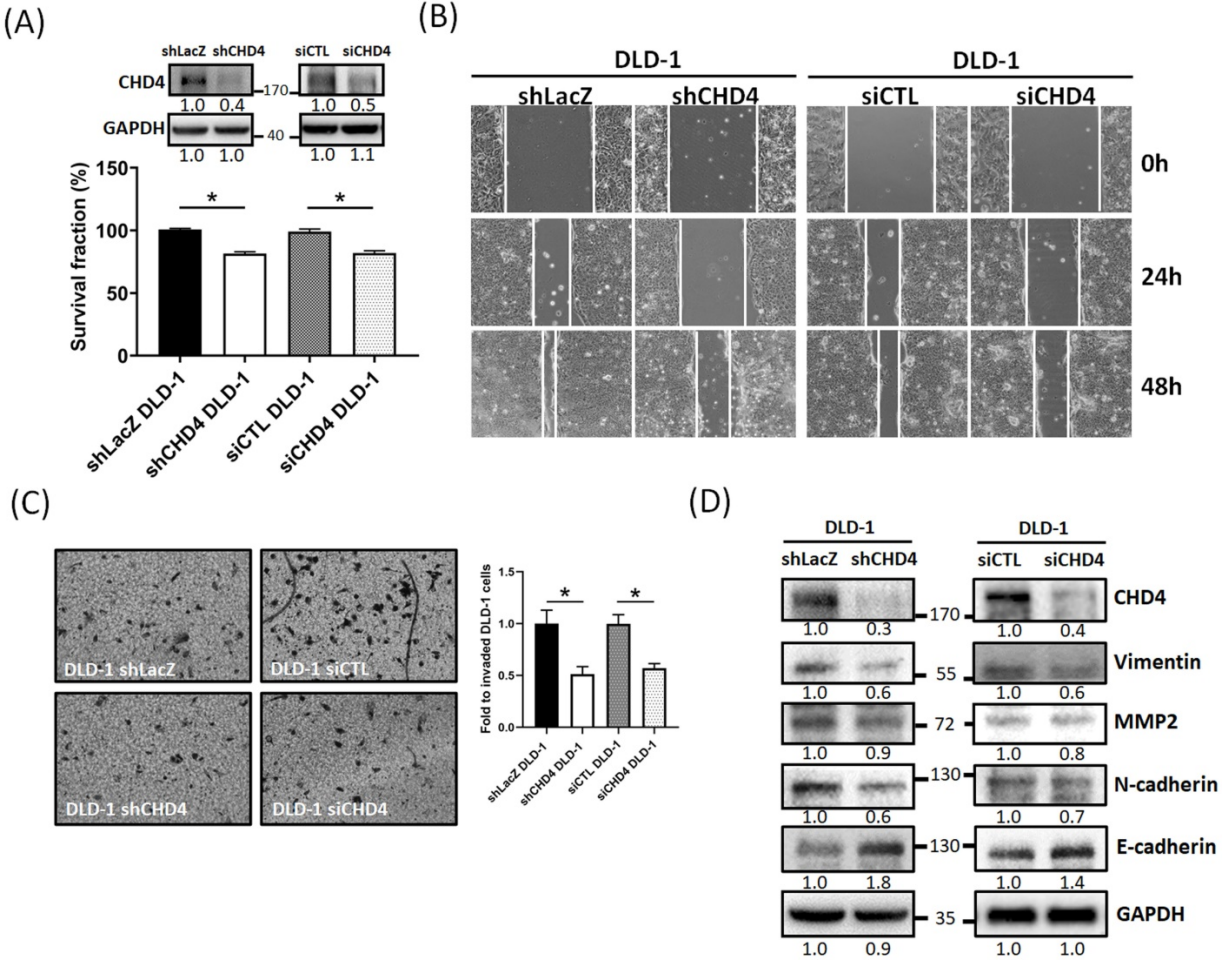
** CHD4 mediates cell motility in CRC cells through the epithelial-mesenchymal transition (EMT)-related mechanism.** (A) Knockdown of *CHD4* in DLD-1 suppresses the cell proliferation. (B) Knockdown of *CHD4* suppresses DLD-1 cell migration. (C) Knockdown of *CHD4* suppresses DLD-1 cell invasion. (D) Knockdown of *CHD4* affects several EMT-associated protein expressions (such as Vimentin, MMP2, N-cadhenin, and E-cadherin) in CRC cells. Data from three independent experiments are used for statistical analysis and * *P* < 0.05.

Our clinical analytical results have shown that CHD4 expression is associated with nodal and metastasis stage and survival. Several prior studies suggested that CHD4 could regulate several malignant characteristics in cancer cells 18, 21, 25, 26, 35, 36. Therefore, we investigated whether CHD4 affected the proliferation and motility of CRC cells. At first, we found that knockdown of *CHD4* using siRNA or shRNA significantly suppressed cell proliferation in DLD-1 cells after 72h culture (Fig. 3A and Fig. S1A). This phenomenon was also seen in other CRC cell lines (Fig. S1B). Next, we found that knockdown of CHD4 using siRNA or shRNA all significantly reduced the number of migrated and invaded in DLD-1 cells (Fig. 3B and C), suggested that CHD4 has effects on regulating cell motility of CRC cells. Our previous study found that CHD4 plays a role in regulating the loss of E-cadherin, one of the epithelial-mesenchymal transition (EMT) protein, then promoting metastatic abilities in TNBC cells 26. In this study, we also found that several EMT-related proteins (such as Vimentin, MMP2, and N-cadherin) were decreased while that of E-cadherin was increased in *CHD4*-depleted DLD-1 cells (Fig. 3D). Hence, these results suggest that CHD4 affected the migratory and invasive abilities of CRC cells through altering several proteins associated with EMT in CRC cells.

### Suppression of CHD4 affects the tumor growth *in vivo*

**Figure 4:**
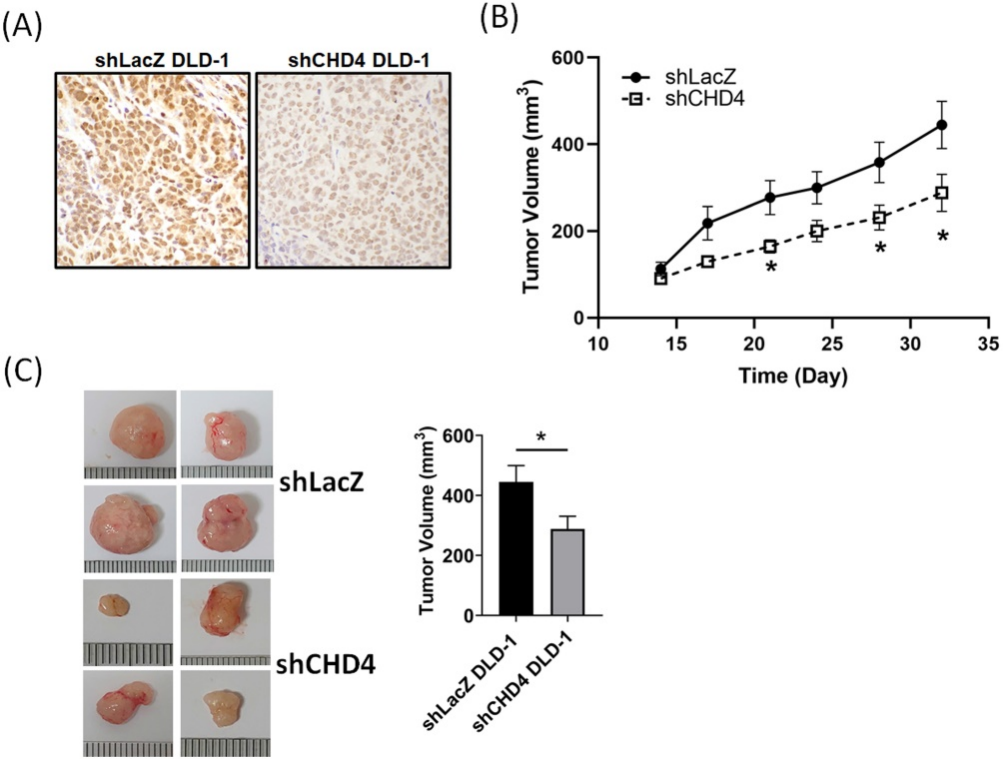
** Suppression of CHD4 affects the CRC tumor cell growth and increases sensitivity to cisplatin.** (A) IHC staining of CHD4 expression in *shLacZ* and *shCHD4* tumor tissues (original 400x magnification). (B) Knockdown of CHD4 reduces the tumor growth rate. (C) Tumor size in *CHD4* depleted DLD-1 tumor is significantly smaller than that in parental DLD-1 tumor. *: *P* < 0.05.

After a series of *in vitro* experiments, we found that CHD4 played important roles in regulating cell proliferation in CRC cells. In order to verify our *in vitro* findings, we next established a xenograft CRC animal model. *shLacZ* and *shCHD4* DLD-1 cells were implanted subcutaneously into the back of the mice. As shown in Fig. 4A, the IHC staining revealed high CHD4 expression in *shLAcZ* and low CHD4 expression in *shCHD4* tumor tissues. In addition, the tumor growth in *shCHD4* DLD-1 cells was slower than that in *shLacZ* DLD-1 cells (Fig. 4B) and the tumor size in *shCHD4* DLD-1 tumor was also significant smaller than *shLacZ* DLD-1 tumor (Fig. 4C). Thus, these results demonstrated that CHD4 could mediate the CRC tumor growth* in vivo*.

### Inhibition of CHD4 increases the sensitivity of CRC cells to cisplatin

**Figure 5:**
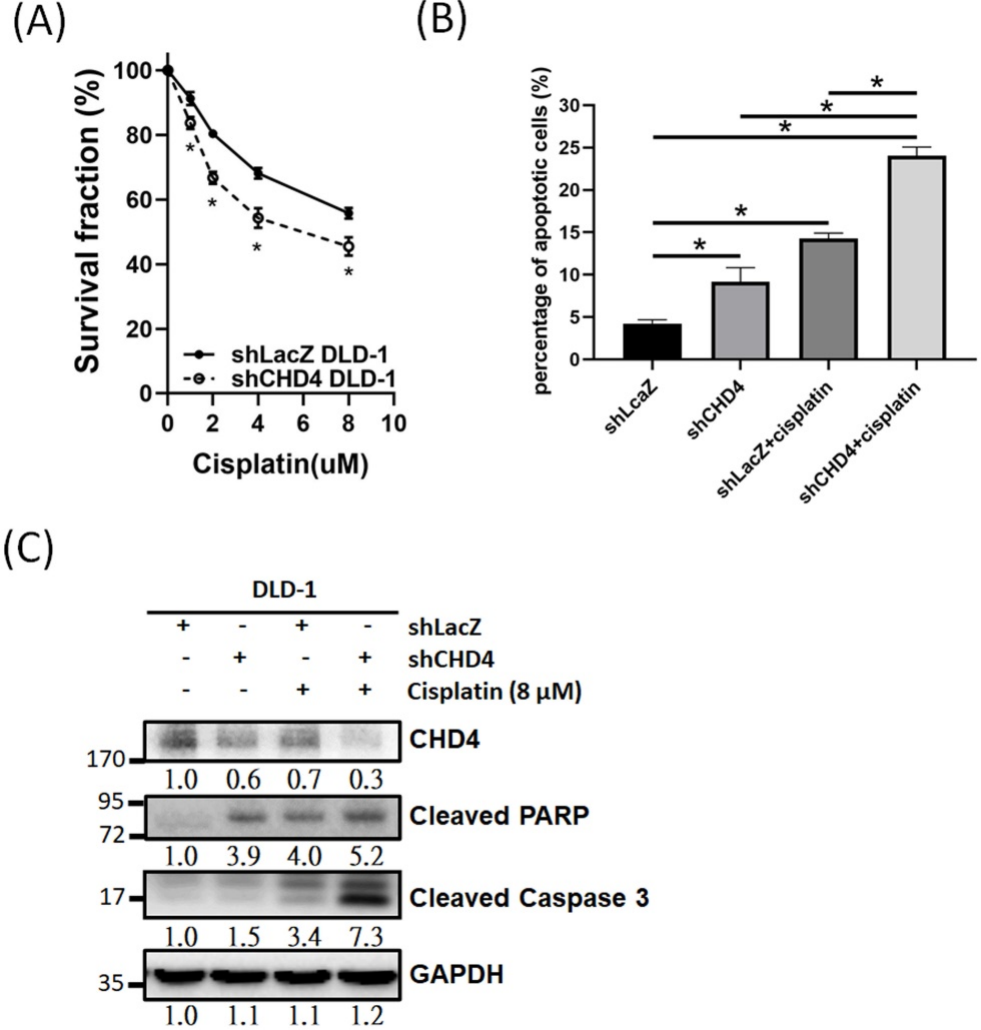
** CHD4 plays a role in regulating drug sensitivity in CRC cells.** (A) Knockdown of *CHD4* in DLD-1 can increase the cytotoxicity of cisplatin. (B) AnV-PI double staining shows that cisplatin induces higher cell death rate through apoptosis in *CHD4* depleted DLD-1 cells. (C) Western blot analysis shows that higher levels of cleaved Caspase3 and PARP in *CHD4* depleted DLD-1 cells were treated with cisplatin. Data from three independent experiments are used for statistical analysis and * *P* < 0.05.

Previous studies have indicated that high CHD4 expression was associated with anti-cancer drug and radiation resistance in several different cancer types 25-27, 37-39. Thus, we investigated the role of CHD4 in regulating the platinum drug sensitivity in CRC cells. As shown in Fig. 5A, knockdown of *CHD4* in DLD-1 cells increased the cytotoxicity induced by cisplatin. Other CRC cancer cells, such as HCT-116 and HT-29, also exhibited the similar sensitizing effect by cisplatin in *CHD4* knockdown cells (Fig. S1C). Moreover, the results of the flow cytometry and western blot analysis also showed that cisplatin could induce higher rate of apoptosis in *shCHD4* DLD-1 cells (Fig. 5B and C). In addition, knockdown CHD4 by siRNA also resulted in similar results (Fig. S1D to F). Therefore, our results indicated that defects in CHD4 resulted in greater platinum drug-induced toxicity in CRC cells, supposed that CRC patients who expressed low CHD4 are more suitable for platinum drug treatment.

### Destabilized NuRD complex can increase sensitivity to platinum drugs in CRC cells

**Figure 6:**
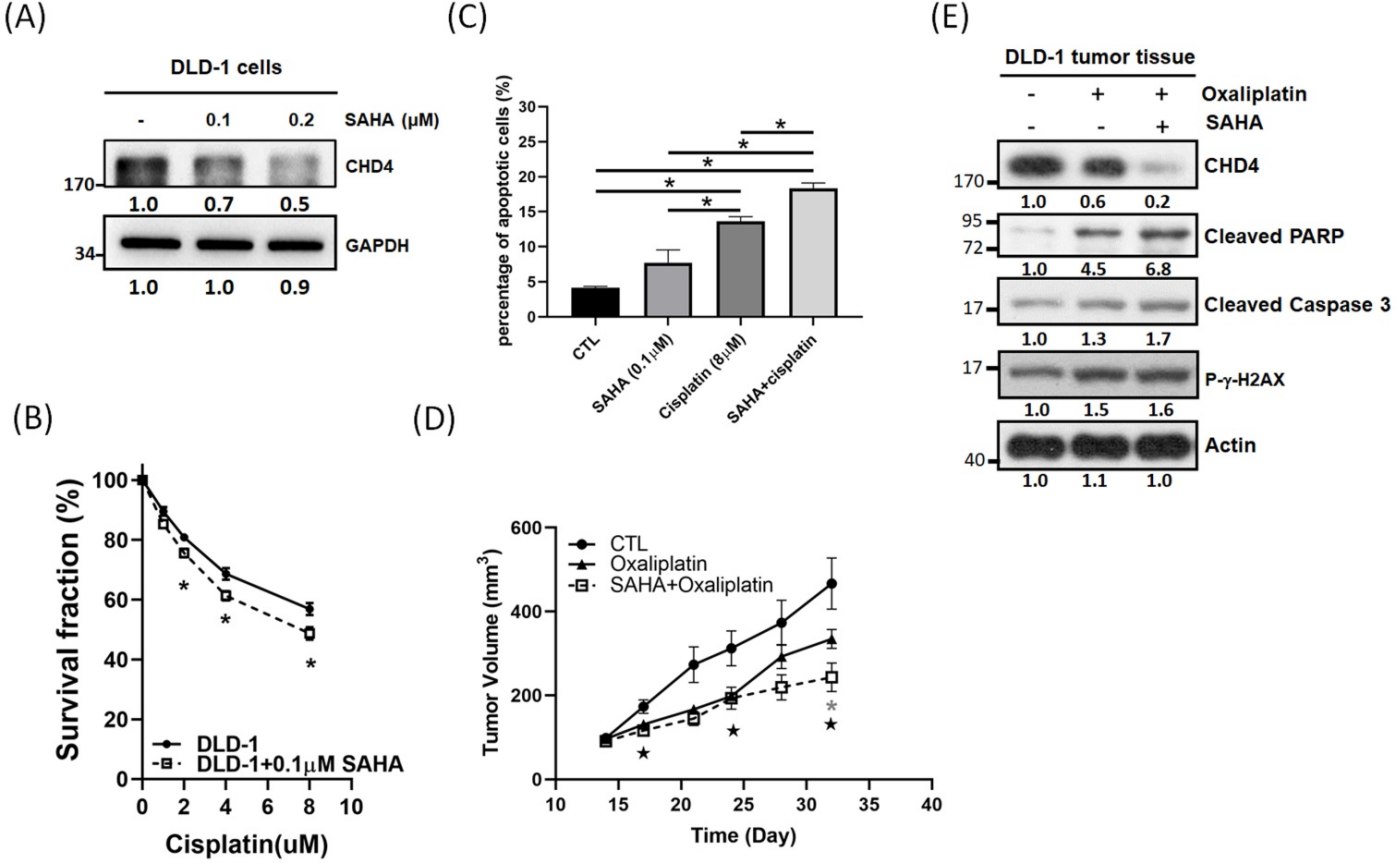
** Impaired of NuRD complex can increase platinum drug sensitivity in CRC cells.** (A) SAHA shows the potential in suppressing CHD4 expression in DLD-1 cells. (B) The combination of cisplatin and SAHA increases the cisplatin cytotoxicity in DLD-1 cells. (C) The combination of cisplatin and SAHA treatment induces more apoptotic cell death than SAHA or cisplatin treatment alone. (D) The combination of oxaliplatin and SAHA inhibits the DLD-1 tumor growth in animal model 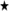: P<0.05 in CTL group vs. SAHA+ Oxaliplatin group; *: P<0.05 in Oxaliplatin group vs. SAHA+ Oxaliplatin group. (E) Western blot analysis shows that the combination of oxaliplatin and SAHA can induce higher rate of apoptotic cell death in DLD-1 tumor model. Data from three independent experiments were used for statistical analysis and *: *P* < 0.05.

Although CHD4 plays important in regulating cell proliferation, motility, platinum drug sensitivity in CRC cells, however, there is no significant inhibitor developed for CHD4 until now. It is well known that CHD4 and HDAC1 are associated with the NuRD complex 15. Thus, we used vorinostat (SAHA), an HDAC inhibitor (HDACi), to destabilize the NuRD complex and to test the role of CHD4 in regulating anticancer drug sensitivity. Surprisingly, our *in vitro* results showed that SAHA could suppress CHD4 expression in DLD-1 cells (Fig. 6A) and other CRC cells (Fig. S1G). MTT assay also showed that the combination of cisplatin and SAHA could also increase the cytotoxicity of cisplatin, and this sensitizing effect is similar to the depletion of *CHD4* then combining with cisplatin (Fig. 6B). In addition, this combination treatment also induced more apoptotic cell death than SAHA or cisplatin treatment alone (Fig. 6C). Our *in vivo* data showed that this combination also inhibited the tumor growth (Fig. 6D) and immunoblotting also showed that the expression of apoptotic markers, such as the cleavages of PARP and Caspase 3, as well as DNA damage markers (γ-H2AX) were increased when combining oxaliplatin and SAHA (Fig. 6E). These results suggested that this combination regulated drug sensitivity could through destabilizing the NuRD complex. Based on our results, this combination treatment can be applied for CRC patients who have high *CHD4* expression. Moreover, the impairment of the NuRD complex can increase the sensitivity to oxaliplatin in CRC cells.

## Discussion

CRC is the third most common cancer in developed countries. Previous studies have indicated that genomic instability, such as chromosomal and microsatellite instabilities, played pivotal roles in CRC development 40. In current clinical practice, surgical resection is the major choice for CRC treatment. Chemo- and radio-therapies are preoperative or postoperative adjuvant therapies for patients with CRC. Although 70-80% CRC tumors can be removed by surgical resection, about half of the patients still exhibit poor response and are resistant to treatments. This results in recurrence and distal metastasis 41, 42. In the past two decades, several investigators have indicated that CRC was a heterogeneous and multistep disease caused by genetic and epigenetic aberrations under microenvironmental influence 13. They also suggested that epigenetic regulation was an important event in colorectal carcinogenesis in addition to genetic alterations.

Several epigenetic mechanisms, such as DNA and histone modification, result in the inheritable silencing of genes without a change in their coding sequence. Epigenetic modulators have become increasingly relevant in clinical practice, especially in applying as diagnostic and prognostic markers as well as novel therapeutic target development 43. Previous studies also indicated that NuRD-mediated chromatin remodeling was necessary for further deposition of epigenetic repressive marks. It was also involved in the progression to advanced stages in different types of cancer 44-48. Among the components of the NuRD complex, CHD4 plays an oncogenic role that initiates and supports TSG silencing in various cancer types 18, 19, 21-24, 26, 27, 36, 39.

In the context of DNA damage, CHD4 is associated with abnormal promoter CpG island DNA methylation and has a key role in initiating and maintaining epigenetic gene silencing 49. This role has been shown to be integral to the oncogenic properties of CHD4 21, 23, 45. High expression of CHD4 has been found to be associated with poor prognosis in various cancer types 18, 21, 23-27, 36. Recent studies also indicated that CHD4 recruited repressive chromatin proteins to help maintain DNA hypermethylation-associated transcriptional silencing of TSGs. High expression of CHD4 in CRC patients was also correlated with radio-resistance 25. In addition, recent studies suggested that CHD4 has a potential to be a prognostic biomarker in triple-negative breast cancer 26, 35 and high CHD4 and 8-hydroxy-deoxyguanosine levels as well as low expression of TSGs were strongly correlated with early disease recurrence and decreased overall survival in CRC patients 21.

In this study, we evaluated the role of CHD4 in regulating several malignant characteristics, such as cell proliferation, motility, drug sensitivity, and tumor growth, through clinical databases, clinical samples,* in vitro* cell models, and *in vivo* animal models. Our results indicated that most CRC patients had higher CHD4 expression in tumor tissues than in normal tissues. Moreover, higher CHD4 expression was correlated with nodal and distant metastasis as well as poorer survival rates. CHD4 also affected cell proliferation and motility by regulating several EMT-related proteins. Knockdown of CHD4 expression might suppress the abovementioned phenomena and increase the platinum drug sensitivity. In animal model, we found that tumor growth in CHD4-depleted cells was lower than that in parental cells, suggesting that high expression of CHD4 promoted tumor growth in CRC.

Although there were no specific inhibitors for CHD4, we used SAHA, one of the HDAC inhibitors, which has potential to destabilize the NuRD complex, in this study. To verify whether using HDAC inhibitors was a viable alternative treatment strategy for CRC patients with high CHD4 expression, SAHA was used in combination with platinum drugs to treat DLD-1 cells and DLD-1 tumors. Our data showed that SAHA inhibited CHD4 expression in CRC cells. Additionally, the combination of SAHA and platinum drugs also suppressed CHD4 protein expression* in vitro* and *in vivo*. This combination also increased the cytotoxicity of platinum drug alone. Previous studies also associated the synergism between HDACi and oxaliplatin with the augmented apoptotic signal. This allowed for the significant dose reduction of anticancer agents 50. However, the mechanisms of downregulation of CHD4 expression by SAHA should be investigated in the further study. Therefore, CHD4 is a potential biomarker for selecting patients to be treated with the combination of HDACis and platinum-drugs. In addition, it is necessary to develop CHD4 inhibitors for CRC patients in the future.

## Conclusions

In summary, we found that CHD4 was overexpressed in tumor tissues in CRC patients. High expression of CHD4 was correlated with poor survival as well as high nodal and distant metastases. In addition, CHD4 regulated tumor growth rate and drug sensitivity. Thus, CHD4 is a potential drug target for treating patients with CRC.

## Supplementary Material

Supplementary figure S1.

## Figures and Tables

**Table 1:** Relationship between CHD4 expression and clinicopathological characteristics of CRC patients (n=83)

Parameters	n	CHD4, n (%)		*P*-value
		Low	High	
Total	83	25 (30.12)	58 (69.88)	
**Tumor stage**				0.4252
T1/T2	7	3 (12.00)	4 (6.90)	
T3/T4	76	22 (88.00)	54 (93.10)	
**Nodal stage**				0.2402
N0	45	16 (64.00)	29 (50.00)	
N1/N2/N3	38	9 (36.00)	29 (50.00)	
**Metastatic stage**				0.0315*
M0	68	24 (96.00)	44 (75.86)	
M1	15	1 (4.00)	14 (24.14)	
**Survival status**				0.0260*
Survival	41	17 (68.00)	24 (41.38)	
Death	42	8 (32.00)	34 (58.62)	

*Statistically significant (P < 0.05).
