# Supplementary material for: CHD4 as an important mediator in regulating the malignant behaviors of colorectal cancer
Source: Int J Biol Sci. 2021 Apr 12;17(7):1660–70. doi: 10.7150/ijbs.56976 (PMC8120460; doi:10.7150/ijbs.56976)
Supplement: Supplementary file 1 — Supplementary figure S1. [file ijbsv17p1660s1.pdf]

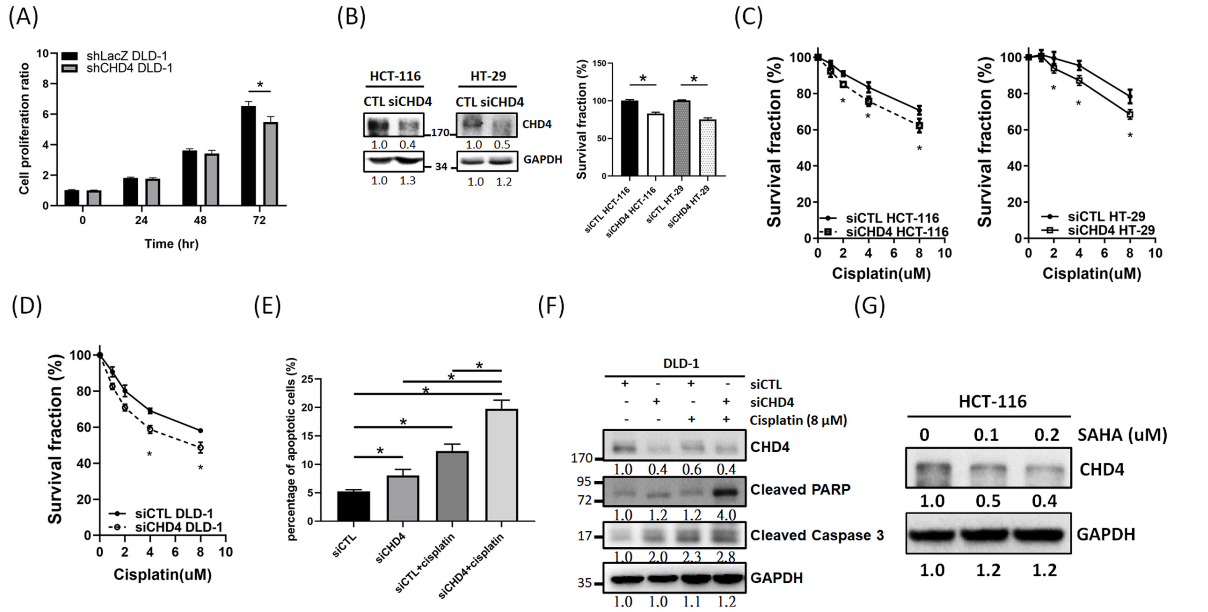

Figure S1. (A) Knockdown of *CHD4* in DLD-1 cells suppresses the cell proliferation. (B) Knockdown of *CHD4* in HCT-116 and HT-29 cells suppresses the cell proliferation. (C) Knockdown of *CHD4* in HCT-116 and HT-29 CRC cells can increase the cytotoxicity of cisplatin. (D) Knockdown of *CHD4* by siRNA in DLD-1 cells can increase the cytotoxicity of cisplatin. (E) AnV-PI double staining shows that cisplatin induces higher cell death rate through apoptosis in *CHD4* depleted DLD-1 cells. (F) Western blot analysis shows that higher levels of cleaved Caspase3 and PARP in *CHD4* depleted DLD-1 cells were treated with cisplatin. (G) SAHA shows the potential in suppressing CHD4 expression in HCT-116 cells. Data from three independent experiments are used for statistical analysis and * $P < 0.05$.
